# On the bibliometric coordinates of four different research fields in Geography

**DOI:** 10.1007/s11192-016-1864-y

**Published:** 2016-02-09

**Authors:** Juan Gorraiz, Christian Gumpenberger, Thomas Glade

**Affiliations:** Library and Archive Services, Bibliometrics and Publication Strategies, University of Vienna, Boltzmanngasse 5, 1090 Vienna, Austria; Department of Geography and Regional Research, University of Vienna, Universitätsstraße 7, 1010 Vienna, Austria

**Keywords:** Citation data sources, Coverage, Google Scholar, Web of Science, Scopus, Geography, Natural sciences, Social sciences, Citation analysis, Publication strategies

## Abstract

This study is a bibliometric analysis of the highly complex research discipline Geography. In order to identify the most popular and most cited publication channels, to reveal publication strategies, and to analyse the discipline’s coverage within publications, the three main data sources for citation analyses, namely Web of Science, Scopus and Google Scholar, have been utilized. This study is based on publication data collected for four individual evaluation exercises performed at the University of Vienna and related to four different subfields: Geoecology, Social and Economic Geography, Demography and Population Geography, and Economic Geography. The results show very heterogeneous and individual publication strategies, even in the same research fields. Monographs, journal articles and book chapters are the most cited document types. Differences between research fields more related to the natural sciences than to the social sciences are clearly visible, but less considerable when taking into account the higher number of co-authors. General publication strategies seem to be established for both natural science and social sciences, however, with significant differences. While in natural science mainly publications in international peer-reviewed scientific journals matter, the focus in social sciences is rather on book chapters, reports and monographs. Although an “iceberg citation model” is suggested, citation analyses for monographs, book chapters and reports should be conducted separately and should include complementary data sources, such as Google Scholar, in order to enhance the coverage and to improve the quality of the visibility and impact analyses. This is particularly important for social sciences related research within Geography.

## Introduction and background

This study is a bibliometric analysis of a highly complex research discipline, namely Geography. Within these different science fields, different publication habits exist, which are explored in this contribution based on four different individual evaluation exercises.

The discipline Geography deals with any observation, analysis, modelling, and theoretical and conceptual approach on any action and development in the space and time domain. It is indeed much more than a “regional” science. It often has a spatial focus, but it also addresses contextual thoughts and developments. Geography includes the study of earth surfaces, landscapes, people, places and the physical and mental environments of humans (Gebhardt et al. 2011b). Hereby, the Geographic domain is either addressed from specific disciplines, for example Spatial Planning or Geomorphology, but also and in particular addresses cross-cutting topics such as Natural Hazards and Risks (Dikau and Pohl 2011), utilization of natural or human resources, or global environmental changes (Glaser and Gebhardt 2011). The computerized assessment and analysis of spatio-temporal pattern has been significantly changed with the introduction and improvement of Geographic Information Systems (GIS) and the introduction of computerized visualization within the traditional field of Cartography. Today, the “Modern Geography” (Gebhardt et al. 2011a) contains fields, which can be attributed to social sciences (e.g. Population -, Economic Geography, Spatial Planning), to natural sciences (e.g. Geomorphology, Geoecology, Biogeography, Climate Geography) and to engineering and IT sciences (e.g. Geoinformation, Cartography). Besides the research focused on these sciences, a traditional focus is also on cross-cutting topics such as climate, environmental and global changes, utilization of resources or natural hazards and risks, since these are research fields which need a multi-disciplinary view on the resulting matters in order to contribute to the significant challenges humans and societies are currently facing. Since not everything can be investigated in one analysis, this analysis focuses on the research areas of Human- and Physical Geography, and does not consider the GIS and IT-domains.

From a bibliometric point of view, Geography is a very challenging discipline. The relation to the natural sciences (e.g. Physical Geography) as well as to the social sciences (e.g. Human Geography) results in an interesting bibliometric analysis of this overarching discipline. These two main directions are perfectly illustrated in the two different editions of the Journal Citation Reports (JCR). They show that the median as well as the aggregate category impact factor is twice as high in the Science than in the Social Science edition.

Although many bibliometric studies have demonstrated the differences between natural and social sciences and the different discipline-dependent publication cultures (e.g. Nederhof 2006; Australian Research Council 2012; Ossenblok et al. 2012; van Leeuwen 2013; Moksony et al. 2014), there is no literature available focusing on specific Geography related contexts.

Consequently, the main research questions of this study are:

- Which are the most usual and most cited publication channels?
- Which data sources are most applicable for Geography bibliometrics? Is Google Scholar indeed a useful complementary data source?
- Are there any bibliometric differences detectable between natural science and human science oriented research fields?

## Data sources and methodology

This study is primarily based on publication data collected for four evaluation exercises performed at the Department for Geography and Regional Research at the University of Vienna. These four exercises have been classed in three parts. The first and second evaluation exercises were related to “Geoecology” and to “Social and Economic Geography”, and comprised twelve and ten individuals or candidates respectively. Both of these analyses were performed during 2013 and are presented in part one. The third exercise was related to “Population Geography and Demography”, comprised nine candidates, was performed in August 2014 and is discussed in part two. The fourth and final exercise discussed in this study was related to “Economic Geography”, comprised nine candidates again, was performed in July 2015 and is presented in part three.

In all exercises, the publication data were delivered directly by the candidates, whose identity has to remain anonymous. Since it can be assumed that the candidates presented all presentations, the publication data are regarded as complete. All bibliometric indicators added to the list of publications were controlled or recalculated in order to enable a correct and comparable analysis (Gorraiz and Gumpenberger 2015a, b). Document types used by the authors in their list of publications were manually reassigned to the following standard groups: Monographs (Books), Book Chapters, Journal Articles, Proceedings Papers, Conferences (including Meeting Abstracts and Talks), Reports (Working Papers), Book Reviews, Edited Books and Journals Issues, and Other Publications (or Miscellaneous).

A clear distinction between “Proceedings Papers” and “Conferences” was not always possible when referring to the provided details in the individual publication lists. However, the resulting possible erroneous classification of entries is regarded as negligible, especially for citation analyses.

The main data source for coverage and citation analyses was “WoS - Core Collection” including the Conference Proceedings and Book Citation Indexes. Since coverage of many human science oriented research fields of Geography in the usual multidisciplinary bibliographic and citation databases (Web of Science, Scopus) is very low and therefore has to be treated carefully within common citation analyses, we have included Google Scholar (GS) as an additional data source in a first explorative attempt (Jacso 2005; Kousha and Thelwall 2007; Meho and Yang 2007).

The analysis in GS was performed by using the GS Citations Profiles. The candidates were invited to create their individual profiles and make them publicly available for at least a couple of weeks. In addition, the tool ‘Publish or Perish’ particularly designed for citation analyses in GS was used to check and amend these profiles (Harzing 2007). In the cases where individual profiles were not available, respective queries have been made in order to assemble a complete data set. It was aimed to be similar and comparable for all candidates in the further analyses.

In spite of the fact that citations were checked and the percentage of self-citations was determined, citation analyses in GS should be taken with a pinch of salt. GS is rather a search engine than a database, and therefore indexing remains non-transparent and documentation is lacking. That is why the analyses were also performed in WoS, including the Cited Reference Search. This means that citations originating from WoS ‘core journals’ were also considered for all document types without any restrictions. Furthermore, Scopus has been utilized as another primary source within the third exercise, corresponding to the following second part (Labbé and Labbé 2013; López-Cózar et al. 2014).

The used publication windows were either the last 10 years or the academic age of each applicant for all four excercises. In order to distinguish individual academic age periods, the year of the first publication activity is always included.

The observed citations window was identical for all applicants for each exercise. It covered the date from the first publication until April–May 2013 for the two first ones (1 and 2), until July–August 2014 for the third one 3, and until July 2015 for the fourth one. Visibility analyses were performed based on the data available from the Journal Citation Reports (JCR), editions 2012 (exercise 1 and 2), 2013 (exercise 3), and 2014 (exercise 4) respectively. The quartiles (Q1 = Top 25 %; Q2 = Top 25–50 %; Q3 = Top 50–75 % and Q4 = Top 75–100 %) were calculated according to the 2-years impact factor (IF) in the corresponding WoS category.

Furthermore, a statistical analysis has also been performed and includes the calculation of the mean, median, standard deviation and the confidence *T* function^1^ (significance level of 0.05 what equates to a confidence level of 95 %). These values are provided at the bottom of the respective tables.

## Results

The results are reported in three parts according to the different excercises. This will avoid any further bias in the interpretation of the data, since the analysis and respective values always refer to the similar period of analysis (Table 1).

**Table 1:** Publication spectrum and WoS coverage according to provided publication list for exercise no.1 (geoecology—12 candidates)

Candidate no.	1st pub year	Books	Edited books/issues	Book chapters	Proceedings and conference papers^a^	Book reviews	Miscellaneous	Journal articles (JA)	% WoS coverage only peer review JA
1	2004	1	0	5 (1)	14 (1)	0	3	28 (24)	85.19 %
2	2002	0	0	6 (1)	35 (3)	0	2	33 (30)	89.66 %
3	1996	13	7	12 (4)	26 (1)	0	0	38 (28)	73.81 %
4	1990	2	4 (2)	25 (6)	17	0	29	17 (11)	64.71 %
5	1998	4	2	1	6 (2)	0	65	75 (61)	80.77 %
6	1998	2	0	8 (2)	55 (2)	0	3	31 (21)	67.86 %
7	2007	4	0	1	41	0	1	35 (33)	94.12 %
8	1994	9	0	16	192	0	0	66 (53)	79.66 %
9	1999	0	0	7	13 (3)	0	5	28 (28)	100.00 %
10	2005	3	0	12	12 (2)	10 (5)	10	18 (11)	60.00 %
11	2002	0	0	5 (1)	70	0	0	28 (18)	63.33 %
12	1994	1	0	2 (1)	8	0	1	51 (51)	100.00 %
Mean		3.25	1.08	8.33	40.75	0.83	9.92	37.33	79.92 %
Median		2.00	0.00	6.50	21.50	0.00	2.50	32.00	80.00 %
Standard deviation		3.81	2.14	6.71	49.45	2.76	18.34	17.18	0.14
Confidence *T*		2.42	1.36	4.26	31.42	1.76	11.65	10.91	0.09

In parentheses, the number of document types indexed in WoS; PY = all years

^a^No distinction

### Part 1: Comparison between exercises 1 (geoecology) and 2 (social and economic geography)

The most important publication document types used by the candidates for both appointments are presented in Tables 2 and 3. The spectrum is much more heterogeneous in the social sciences, where articles in peer-reviewed journals are not always the most common publication channel. Furthermore, many publications in the social science domains of Geography are published in German, since these are the dissemination products which reach the targeted audience.

**Table 2:** Publication spectrum and WoS coverage according to provided publication list for exercise no. 2 (social and economic geography—10 candidates)

Candidate no.	1st pub year	Books	Edited books/issues	Book chapters	Proceedings and conference papers^a^	Book reviews	Miscellaneous	Journal articles (JA)	% WoS coverage only peer review JA
1	1999	3	2	8 (1)	2	8	50	72 (35)	47.17 %
2	2002	3	11	21	5 + 56^a^	0	0	16 (8)	50.00 %
3	1991	7	0	19 (1)	87^a^	0	13	37 (18)	48.39 %
4	1993	3	0	17 (2)	67^a^	19 (9)	44	46 (24)	52.50 %
5	1994	7	2	16	2 + 34^a^	0	9	31 (17)	53.85 %
6	2005	3	5	15	42^a^	0	5	15 (4)	27.27 %
7	1990	3	11	58	*4*	10	14	35 (22)	62.50 %
8	2005	1	1	5	*40* ^a^	0	9	20 (7)	35.29 %
9	2004	3 (1)	0	21 (7)	*10* ^a^	2	10	16 (11)	72.73 %
10	2000	3	1	17	72^a^	0	49	22 (11)	50.00 %
Mean		3.60	3.30	19.70	42.10	3.90	20.30	31.00	49.97 %
Median		3.00	1.50	17.00	41.00	0.00	11.50	26.50	50.00 %
Standard deviation		1.80	4.10	13.69	28.32	6.14	18.35	16.93	0.12
Confidence *T*		1.29	2.93	9.79	20.26	4.39	13.13	12.11	0.09

In parentheses, the number of document types indexed in WoS; PY = all years

^a^No distinction

**Table 3:** Visibility (Q1 and  %Q1) and citation analysis in WoS for exercise 1 (geoecology—12 candidates)

Candidate no.	1st pub year	Publications			# Authors per paper	Citations ARPP			h-Index	% Self-citations	Q1	% Q1
		Total	ARPP	per Y		Sum	per P	Max				
1	2004	25	25	2.78	6.36	147	5.88	28	7	16.22 %	16	69.57 %
2	2002	28	28	2.80	4.93	181	6.46	36	7	24.31 %	14	87.50 %
3	1996	29	26	2.60	4.83	249	9.58	31	10	19.05 %	14	53.85 %
4	1990	11	7	0.70	2.73	29	4.14	21	3	12.50 %	5	100.00 %
5	1998	49	48	4.80	5.57	458	9.54	42	12	30.07 %	34	72.34 %
6	1998	18	18	1.80	3.72	180	10.00	44	7	7.78 %	8	53.33 %
7	2007	32	32	5.33	5.53	428	13.38	155	12	21.26 %	20	62.50 %
8	1994	31	29	2.90	5.06	598	21.36	110	15	7.18 %	29	93.55 %
9	1999	17	17	1.70	4.94	317	18.65	102	7	4.73 %	6	42.86 %
10	2005	16	11	1.38	2.94	40	3.64	24	3	10.00 %	2	14.29 %
11	2002	16	16	1.60	4.38	129	8.06	21	8	15.50 %	9	60.00 %
12	1994	36	26	2.60	4.69	294	11.31	44	12	17.06 %	32	91.43 %
Mean		25.67	23.58	2.58	4.64	254.17	10.17	54.83	8.58	15.47 %	15.75	66.77 %
Median		26.50	25.50	2.60	4.88	215.00	9.56	39.00	7.50	15.86 %	14	66.03 %
Standard deviation		10.30	10.39	1.29	1.02	165.86	5.20	41.42	3.55	0.07	10.417	0.24
Confidence *T*		6.54	6.60	0.82	0.65	105.38	3.31	26.31	2.25	0.05	6.6189	0.15

PY = 2003–2012

*ARPP* articles, reviews and proceedings papers

The mean and median number of publications in Books, Edited Books and especially Book Chapters is higher in Social and Economic Geography than in Geoecology. Tables 2 and 3 also show that almost twice as many publications were produced via other channels (see Miscellaneous) in Social and Economic Geography. This reflects the already addressed importance of this research field to reach the targeted German-speaking audience.

*Miscellaneous* mostly comprised of Reports and Working Papers in both appointments. Therefore this document type was considered separately in the second part of the study.

In exercise 2, other document types such as Films, Policy Briefs, Newspapers and Special Issues were mentioned, albeit only individually, and counted under the category *Miscellaneous*. This can again be explained that in Social Science research fields, other means of dissemination of research results play an important role for making scientific research accessible. For two candidates (one in each exercise), articles in *Other* (non-scientific or non-peer-reviewed) journals were also assigned to the group *Miscellaneous*.

Concerning the coverage in WoS, both tables corroborate the still low inclusion of Books and Book Chapters in both editions of the Book Citation Index (Gorraiz et al. 2013). For articles in peer-reviewed journals, the WoS coverage in exercise 1 ranges from 60 to 100 %. This trend has been constantly increasing in the last 10 years until having reached a value of almost 90 % for all candidates. In exercise 2 the coverage was lower, with values from 30 to 60 %. However, a similar trend could also be observed, despite of the fact that the increase was not so steep. This reflects the observed situation. Within natural science oriented Geography (= exercise 1), it is more common to publish the research results in English peer-reviewed journals. However, it has been recognized by many researchers within the various fields of Human Science related Geography, that they have to increase their participation in the English research journals. As the numbers show, the numbers of respective publications raise, as well as the increased trend is visible in both science domains.

Tables 3 and 4 show the results of the visibility and citation analyses performed for both exercises. Only publications indexed in WoS in the last ten complete years (2003–2012) were considered. These results corroborate the higher number of publications and citations in the discipline related to the natural sciences (approximately twice as many). Nevertheless the number of co-authors and the percentage of self-citations, which is almost twice as high in the natural sciences, need to be taken into account as well. By doing so, no considerable difference can be observed.

**Table 4:** Visibility (Q1 and  %Q1) and citation analysis in WoS for exercise 2 (social and economic geography—10 candidates)

Candidate no.	1st Pub Year	Publications			# Authors per Paper	Citations ARPP			h-Index	% Self-citations	Q1	% Q1
		Total	ARPP	per Y		Sum	per P	Max				
1	1999	22	15	1.50	1.14	122	8.13	53	6	11.02 %	12	60.00 %
2	2002	7	4	0.40	2.00	22	5.50	10	3	9.09 %	0	0.00 %
3	1991	12	9	0.90	1.75	352	39.11	94	7	3.13 %	9	81.82 %
4	1993	23	12	1.20	2.61	134	11.17	76	6	13.41 %	7	31.82 %
5	1994	13	9	0.90	2.23	76	8.44	34	4	3.13 %	3	23.08 %
6	2005	4	3	0.38	1.00	3	1.00	2	1	0.00 %	0	0.00 %
7	1990	18	13	1.30	2	36	2.77	11	3	24.32 %	3	18.75 %
8	2005	7	6	0.75	2.57	48	8.00	17	4	8.33 %	1	14.29 %
9	2004	17	14	1.56	1.82	259	18.50	149	5	8.33 %	7	70.00 %
10	2000	8	7	0.70	1.13	53	7.57	40	3	9.26 %	1	12.50 %
Mean		13.10	9.20	0.96	1.82	110.50	11.02	48.60	4.20	9.00 %	4.3	31.22 %
Median		12.50	9.00	0.90	1.91	64.50	8.07	37.00	4.00	8.71 %	3	20.91 %
Standard deviation		6.33	3.99	0.40	0.55	106.96	10.39	43.90	1.72	6.40 %	3.98	0.28
Confidence *T*		4.53	2.86	0.29	0.40	76.51	7.43	31.40	1.23	4.58 %	2.84	0.20

PY = 2003–2012

*ARPP* articles, reviews and proceedings papers

These numbers reflect again the differences in the social and natural science oriented Geography. With the natural science domains, it is much more common to publish also many papers during the research process. In particular if this research is embedded in third-party funded research projects, a respective output also in terms of research publications in peer-reviewed journals is required. Although this is also true for social science, the respective research directions in Geography traditionally prefer to publish the results in larger contributions, thus summarizing findings in book chapters or monography rather than publishing also “smaller” research findings individually. Since one has to cite previously published work, the candidates in natural science show higher numbers in self-citations than the candidates in social science research.

The visibility analysis (number of Q1—Journal Articles) shows that publishing in top journals with high impact factor result in a much higher visibility for the appointment related to natural sciences than for the one related to the social sciences. As already previously explored, this can be related to the different value of publishing in peer-reviewed journals, which is much more important for natural than for social science related research.

Finally, Tables 5 and 6 show that the citation differences, according to the aggregate impact factor of the main WoS category, are higher for exercise 1 than for 2. For example, the WoS category “Ecology” has an aggregate impact factor of 3.095, which is almost three times higher than for “Geography” with 1.469.

**Table 5:** First and second research field according to WoS Categories for exercise 1 (geoecology—12 candidates)

Candidate no.	First research field (2003–2012)			Second research field (2003–2012)
	WoS category	IF median	IF aggregate	WoS category
1	Ecology	1.828	3.095	Environmental sciences
2	Remote sensing	1.079	1.845	Geosciences, multidisciplinary
3	Water resources	1.096	1.803	Geosciences, multidisciplinary
4	Water resources	1.096	1.803	Geosciences, multidisciplinary
5	Soil science	1.530	1.780	Geosciences, multidisciplinary
6	Ecology	1.828	3.095	Forestry/soil science/environm. sci
7	Ecology	1.828	3.095	Forestry/plant sciences
8	Geosciences, multidisciplinary	1.332	2.176	Geography, physical
9	Geosciences, multidisciplinary	1.332	2.176	Geography/water resources
10	Geography, physical	1.481	2.206	Geography/remote sensing
11	Water resources	1.096	1.803	Soil Sciences/environmental sci
12	Geochemistry and geophysics	1.474	2.319	Oceanography/geosciences, multi

**Table 6:** First and second research field according to WoS Categories for exercise 2 (social and political geography—10 candidates)

Candidate no.	First research field (2003–2012)		Second research field (2003–2012)
	WoS category	IF aggregate 2012	WoS category
1	Geography	1.469	Industrial relations and labor
2	Geography	1.469	Environmental sciences
3	Geography	1.469	Economics; management
4	Geography	1.469	Environmental studies; economics
5	Geography	1.469	Economics
6	Geography	1.469	Geography, physical
7	Geography	1.469	Urban studies
8	Geography	1.469	Environmental studies and sciences
9	Economics	1.148	Geography; planning and development
10	Geography	1.469	Economics

Both tables demonstrate, that the natural science domains contribute significantly to highly specialised international journals in order to place the respective research results in the relevant research community. The same is indeed true for social science publications; however, the respective applicants in these exercises tend to publish in Geography related journals and not so much in journals of other disciplines with higher median category impact factors. Nevertheless, it can be recognized that the researchers have become aware of this situation, and that most recently the numbers of publications in interdisciplinary journals with higher IF have increased.

### Part 2: Results obtained in exercise 3 (demography and population geography)

Within this evaluation exercise, applicants were invited to create their individual GS Citations profiles and make them publicly available for at least a couple of weeks. Out of nine applicants six created their GS Citation Profile, two refused to do so, and one followed the invitation, but the profile was delivered in an incomplete status. Therefore, the tool ‘Publish or Perish’,, was then used for collecting the missing and checking the provided data (see “Data sources and methodology”).

According to the WoS Categories, the candidates were publishing mainly in Geography, Demography and Urban Studies, categories with very similar aggregate and median impact factors according to the JCR Edition 2014.

**Fig. 1:**
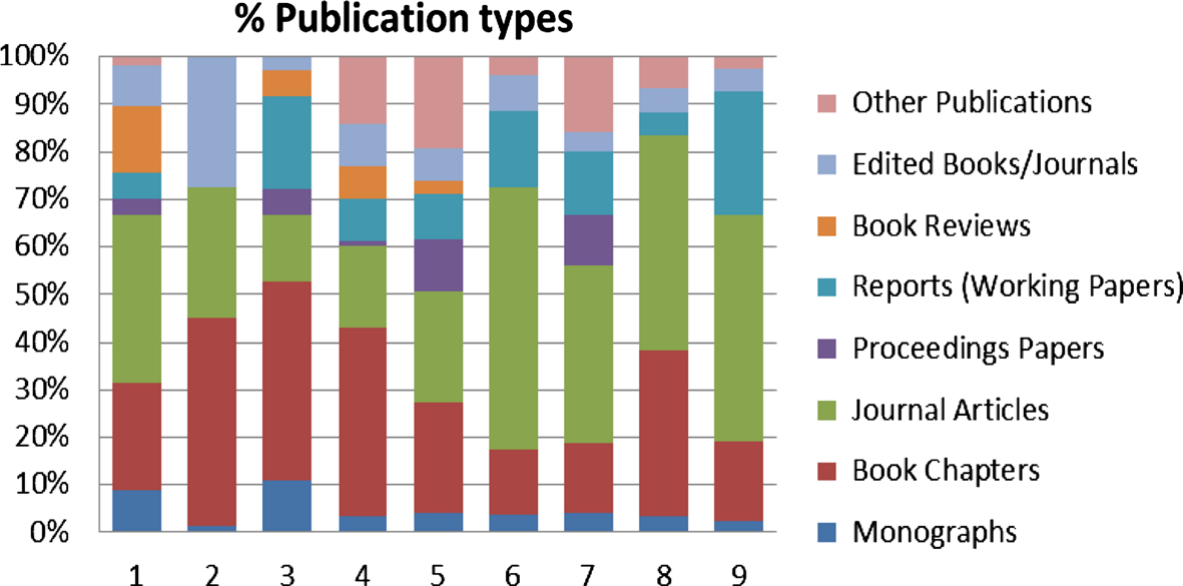
Percentage of publication types for each candidate (Exercise 3)

Figure 1 represents the publication activity for each scientist according to the most relevant publication types. The data are based on the list of publications submitted by the candidates. The table reveals that some candidates published their geography related research more in journals which are summarized within the category “Geography”, whilst others publish rather in journals which are categorized to other specific disciplines, e.g. “Urban Studies”. Besides this different publication strategy, also the assignment of specific journals in the different data bases varies. Consequently, the usage of such categories has to be handled with care, since it these are also dependent on the different internal classification schemes of the data bases.

Book chapters and journal articles are the most frequent publication types. This confirms the already provided statement, that in the social science domains of Geography, it is much more common to distribute the research findings in these means of dissemination. The results hint at very heterogeneous and individual publication strategies based on the analysed publication types. However, a clear trend towards an increased publication within scientific journals was observed in all exercises.

The next three sections contain coverage and citation analyses performed in the three considered data sources. Citation analyses include following indicators for each data source: (1) number of indexed publications; (2) percentage of publications covered according to the provided publication list; (3) number of cited documents; (4) total number of citations; (5) number of citations per cited publication; (6) maximum number of citations attracted by a publication; (7) total h-index and (8) i-index (number of publications with more than 10 citations). The percentage of self-citations in GS was only calculated if the number of citations was of sufficient significance.

Table 7 shows the percentage of coverage in GS for each publication type. In order to distinguish individual academic age periods, the year of the first publication activity has always been included. Monographs (Books) and Edited Books or Issues are very well covered, most likely due to the inclusion of Google Books (Kousha and Thelwall 2009).

**Table 7:** Coverage (%) in GS for each publication type (Exercise 3)

Candidate no.	1	2	3	4	5	6	7	8	9	Mean	Median	Standard deviation	Confi-dence *T*
GS profile	Yes	Incom-plete	Yes	No	No	Yes	Yes	Yes	Yes				
Total (excl. conferences)	58	73	36	121	73	80	75	60	42	68.67	73	23.36	17.96
Total pub (excl. Conf)	44.83 %	52.05 %	44.44 %	57.02 %	35.62 %	72.50 %	77.33 %	68.33 %	97.62 %	61.08 %	57.02 %	0.18	0.14
Monographs	60.00 %	100.00 %	50.00 %	75.00 %	100.00 %	100.00 %	100.00 %	100.00 %	100.00 %	87.22 %	100.00 %	0.19	0.15
Book chapters	16.67 %	12.50 %	40.00 %	56.25 %	35.29 %	45.45 %	90.91 %	42.86 %	100.00 %	48.88 %	42.86 %	0.28	0.22
Journal articles	85.00 %	50.00 %	60.00 %	71.43 %	41.18 %	81.82 %	82.14 %	100.00 %	100.00 %	74.62 %	81.82 %	0.20	0.15
Proceedings papers^a^	n.a.	n.a.	50.00 %	n.a.	25.00 %	n.a.	100.00 %	n.a.	n.a.	58.33 %	50.00 %	0.31	0.77
Reports	66.67 %	n.a.	28.57 %	54.55 %	28.57 %	46.15 %	60.00 %	33.33 %	90.91 %	51.09 %	50.35 %	0.20	0.16
Book reviews	n.a.	n.a.	50.00 %	25.00 %	100.00 %	n.a.	n.a.	n.a.	n.a.	58.33 %	50.00 %	0.31	0.77
Edited books/journals	20.00 %	70.00 %	100.00 %	81.82 %	80.00 %	83.33 %	100.00 %	66.67 %	100.00 %	77.98 %	81.82 %	0.24	0.18
Other publications	n.a.	n.a.	n.a.	41.18 %	n.a.	33.33 %	41.67 %	n.a.	100.00 %	54.04 %	41.42 %	0.27	0.43
1st year	1998	1994	1998	1995	1999	1992	1999	1995	2002	1996.89	1998	2.92	2.25

*n.a.* not applicable

^a^No distinction

The coverage of Journal Articles is also much higher in GS than in WoS or Scopus (see a more detailed insight in Gorraiz and Gumpenberger 2015a, b). This might also refer to the fact, that WoS and SCOPUS include much more natural and engineering science journals than social science ones. This bias within the data base is not so evident within GS, since this database contains much more extensively also other means of publications.

The high coverage of Reports (Working Papers) is remarkable as well. Again, in the social science domains of Geography, the results have to be presented also to the specific groups on stake, and therefore, the preferred place of publishing these findings are often reports and working papers. Chapters in Books are not that well covered, but this is probably due to incidental incorrect citations.

Table 8 shows the results of the coverage and citation analyses performed in WoS, including the Cited Reference Search, in Scopus, and in GS. The higher coverage scores in WoS compared to Scopus are due to the inclusion of the Cited Reference Search, which enables the retrieval of citations not only restricted to journal articles and book indexed in WoS, but also from other books, reports and other document types cited by the core journals in WoS.

**Table 8:** Coverage and citation analysis in the three data sources for each candidate (Exercise 3)

Data Source	Citation analysis	Mean	Median	Standard deviation	Confidence *T*
Google Scholar	Total Pub (excl. Conf)	45.00	44	17.29	13.29
	% covered in GS	61.08 %	57.02 %	0.18	0.14
	# cited documents	30.78	23	16.42	12.62
	Total Citations	355.44	142	334.29	256.95
	% Self-citations	13.65 %	14.52 %	0.05	0.04
	Citations/Cited Pub	9.28	6.625	5.17	3.97
	Maximum Citations	69.78	49	58.89	45.26
	h-index	9.22	8	4.87	3.74
	i10	9.67	5	8.67	6.66
WoS + Cited Ref Search	Total Pub (excl. Conf)	21.67	15	12.95	9.96
	% covered in WoS + CRS	25.80 %	17.24 %	0.17	0.13
	# cited documents	19.11	12	12.67	9.74
	Total Citations	87.89	39	126.54	97.27
	Citations/Cited Pub	3.35	2.73	2.41	1.85
	Maximum Citations	16.33	10	15.43	11.86
	h-index	4.33	4	3.09	2.38
	i10	2.22	1	4.24	3.26
Scopus	Total Pub (excl. Conf)	11.67	10	7.41	5.69
	% covered in Scopus	16.87 %	15.52 %	0.09	0.07
	# cited documents	7.89	7	6.37	4.89
	Total Citations	65.22	27	114.27	87.84
	Citations/Cited Pub	5.02	4.4	4.26	3.27
	Maximum Citations	17.56	11	17.15	13.18
	h-index	3.22	2	2.97	2.29
	i10	2	1	3.94	3.03

Table 8 confirms that the values of the main citation indicators (number of citations, citations per cited publication and h-index) are different in absolute values in GS, WoS and Scopus, but are comparable in terms of relative values. This observation refers again to the better coverage of social science related research in GS rather than in WoS and Scopus.

However, spearman correlations performed for these indicators (number of citations, citations per cited publication and h-index) in the three data sources (WoS, Scopus and Google Scholar) were very strong (varying from 0.8 to 0.95).

A detailed coverage and citation analysis for the three most cited document types in GS, namely Monographs, Book Chapters and Journal Articles is presented in Table 9.

**Table 9:** Detailed citation analysis GS for each candidate and the three most cited publication types (Exercise 3)

Statistical data	Monographs in GS								
	Items				Citations				
	**#** Total	# Not list	# Cited	% cited	# Total	Mean^a^	# Max	# Self	% Self
Mean	2.44	0.11	2.22	92.59 %	53.44	21.17	35.44	4.00	9.76 %
Median	3	0	2	100.00 %	20	12.5	17	3	4.02 %
Stand. deviation	0.68	0.31	0.63	0.14	72.07	23.11	47.37	3.46	0.13
Confidence *T*	0.53	0.24	0.48	0.11	55.40	17.76	36.41	2.66	0.10

Statistical data	Journal articles in GS								
	Items				Citations				
	# Total	# Not list	# Cited	% cited	# Total	Mean^a^	# Max	# Self	% Self
Mean	17.56	0.00	13.44	74.20 %	192.22	10.06	44.00	28.78	11.22 %
Median	17	0	12	70.59 %	68	6.25	20	7	14.13 %
Stand. deviation	9.73	n.a.	8.85	0.14	263.29	7.07	44.25	40.58	0.08
Confidence *T*	7.48	n.a.	6.80	0.11	202.38	5.44	34.02	31.20	0.06

Statistical data	Book chapters in GS								
	Items				Citations				
	# Total	# Not list	# Cited	% cited	# Total	Mean^a^	# Max	# Self	% Self
Mean	8.67	0.22	6.44	68.77 %	55.67	6.21	17.67	9.00	23.04 %
Median	6	0	4	66.67 %	12	5.5	8	5.5	25.00 %
Stand. deviation	6.88	0.42	6.85	0.26	93.01	4.43	24.84	8.35	0.19
Confidence *T*	5.29	0.32	5.26	0.20	71.50	3.41	19.10	6.42	0.15

^a^Cited mean, # Citations/cited item

*n.a.* not applicable

The results show that the most cited publication types differ for each candidate on an individual level. As a consequence, a separate citation analysis of these publication types is then recommended for evaluation purposes. In general, Monographs have the highest percentage of cited items (more 90 %), followed by Journal Articles (~75 %) and Book Chapters (~70 %). Moreover Monographs attract the highest average of citations (cited mean), whereas Journal Articles contribute most to the total impact (total number of citations) for all the individuals. Book Chapters receive almost twice as many self-citations than Journal Articles, and Monographs show the lowest percentage of self-citations. The number of publications covered in GS and not included in the candidates’ publication lists remained very low and were irrelevant for the citation analysis.

### Part 3: Results obtained in exercise 4 (economic geography)

Apart from the research focus of candidate no. 8, who was rather related to Economics than to Geography, no considerable differences were perceived, neither in the WoS category nor in the value of the aggregate impact factor (1.28 versus 1.66).

**Fig. 2:**
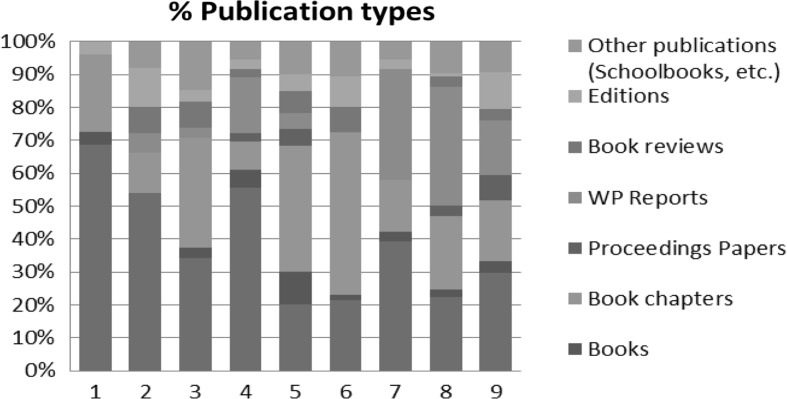
Percentage of publication types for each candidate (Exercise 4)

Figure 2 represents the publication activity for each scientist according to the most relevant publication types. The data are based on the list of publications submitted by the candidates. Like for the previously discussed appointments (see Fig. 1), the results hint at very heterogeneous and individual publication strategies based on the analysed publication types. Some researchers prefer to publish their research within high ranking journal (incl. Q1-journals), other do not consider journals as an important publication channel and prefer to publish in reports and book chapters. The already extensively explored different publication philosophies and strategies are clearly visible.

Table 10 shows the percentage of coverage in WoS, WoS Cited Reference Search and GS for each publication type.

**Table 10:** Coverage (%) in WoS, WoS cited reference search and GS for each publication type (Exercise 4)

Data source	Publication type	Mean	Median	Standard deviation	Confidence *T*
WoS Core Collection	Journal articles	61.31 %	61.90 %	0.17	0.13
	Monographs	6.25 %	0.00 %	0.17	0.14
	Book chapters	14.09 %	0.00 %	0.26	0.20
	Proceedings papers	25.00 %	0.00 %	0.43	0.69
	Book reviews	78.88 %	75.00 %	0.20	0.15
	Total items	21.11	20	9.46	7.27
	% Total items Coverage	32.27 %	28.17 %	0.15	0.12
WoS Cited Reference Search	Journal articles	72.99 %	70.00 %	0.17	0.13
	Monographs	77.08 %	75.00 %	0.40	0.33
	Book chapters	32.96 %	31.03 %	0.21	0.16
	Proceedings papers	106.25 %	66.67 %	0.97	1.54
	WP Reports	12.61 %	0.00 %	0.16	0.15
	Book reviews	14.52 %	0.00 %	0.23	0.21
	Editions	53.70 %	0.00 %	1.07	0.83
	Other publications	66.66 %	41.67 %	0.62	0.51
	Unassigned	1.67	2	1.56	1.20
	% Unassigned	4.51 %	3.77 %	0.05	0.04
	Total items	38.22	36	17.79	13.67
	% Total items coverage	53.69 %	50.70 %	0.22	0.17
Google Scholar	Journal articles	91.13 %	93.33 %	0.13	0.10
	Monographs	100.00 %	100.00 %	0.61	0.51
	Book chapters	66.58 %	66.67 %	0.27	0.21
	Proceedings papers	56.25 %	50.00 %	0.30	0.47
	WP reports	52.00 %	37.50 %	0.27	0.25
	Book reviews	61.94 %	50.00 %	0.34	0.31
	Editions	33.33 %	33.33 %	0.29	0.23
	Other publications	151.27 %	139.29 %	1.11	0.93
	Unassigned	4.11	3	4.82	3.70
	% Unassigned	6.41 %	5.05 %	0.06	0.05
	Total items	58.78	55	27.23	20.93
	% Total items coverage	78.27 %	76.06 %	18.76 %	14.42 %

Monographs (Books) are completely covered in GS (100 %), whereas coverage is less complete in WoS Cited Reference Search (~75 %) and almost zero in WoS Core Collection including both Book Citation Indices. The good results in GS probably originate from the inclusion of Google Books (Kousha and Thelwall 2009) as already noted in the previous section. Similar trends are reported for the other publication types. However, the percentage of book chapters covered in GS is still low (around 55 %) but high in comparison to WoS (14.1 %) and WoS Cited Reference Search (32.9 %). Less than five percent of the publications retrieved in WoS Cited Reference Search and approximately 6 % in Google Scholar could not be assigned to one specific publication type due to missing bibliographic information. It is assumed, that these related to the so-called “grey-literature” (publications, which have been published as internal reports and papers, without an ISBN or ISSN). Furthermore, both data sources, WoS Cited Reference Search and GS included other publications not mentioned by the candidates in their publication lists. These are responsible for values > 100 % given in Table 10. The bias between the two data bases of WoS’s and GS are again clearly visible.

Since the ranking of journals in Q1 to Q4 journals is getting more and more important, this classification has also been performed for both number and percentage of publications in top journals according to their impact factor (IF) in WoS (see Table 11). The Impact Factor data are based on Journal Citation Reports (JCR) Editions 2014. The quartiles (Q1 = Top 25 %, Q2 = Top 25–50 %, Q3 = Top 50–75 %, Q4 = Top 75–100 %) were calculated according to IF in the corresponding Web of Science category.

**Table 11:** Number and percentage of publications in Q1-Journals in WoS (Exercise 4)

Candidate no	1	2	3	4	5	6	7	8	9	Mean	Median	Standard deviation	Confi-dence *T*
First PY	1995	1992	1993	2005	2001	1992	2001	2002	2001				
# Q1	19	17	3	6	2	2	7	10	4	7.78	6.00	6.00	4.61
# Q2	1	6	1	1	1	3	4	5	2	2.67	2	1.83	1.40
# Q3	0	0	0	3	0	0	0	0	0	0.33	0	n.a.	n.a.
# Q4	3	1	5	2	8	21	6	1	1	5.33	3	6.02	4.63
% Q1	82.61 %	70.83 %	33.33 %	50.00 %	18.18 %	7.69 %	41.18 %	62.50 %	57.14 %	47.05 %	50.00 %	0.23	0.18

These results reveal clear publication strategies concerning journal articles. Except candidates 3, 5, 6 and 7, most of the candidates publish predominantly in top Q1 journals according to their impact factor. This reveals and confirms the already previously mentioned observation, that it is recognized also within the social science fields of Geography to publish the research results in highly visible journals.

Table 12 provides the results of the co-authorship analysis and shows very different publication habits. Authors with a higher number of co-authors are usually not only the most productive ones, but also the ones who attract the highest impact scores.

**Table 12:** Co-authorship analysis in WoS Core Collection (Exercise 4)

Number of co-authors in Web of Science				
Candidate no.	Mean	Maximum	# Single-authored	% Single-authored
1	1.24	19	18	62.07 %
2	1.50	19	12	42.86 %
3	0.33	2	9	75.00 %
4	0.86	2	6	42.86 %
5	0.27	1	8	72.73 %
6	0.75	5	23	71.88 %
7	1.30	4	4	20.00 %
8	1.51	3	8	22.86 %
9	0.67	2	4	44.44 %
Mean	0.94	6.33	10.22	50.52 %
Median	0.86	3	8	44.44 %
Standard deviation	0.45	6.86	6.09	0.20
Confidence *T*	0.28	4.36	3.87	0.13

It must be stressed, however, that a high number of co-authors can be interpreted in many ways (strong cooperation but also undeserved or gift authorship) and needs in each particular case a deeper analysis basing on the role played by each author. Therefore, many journals are already asking for the precise role of each co-author (see for example, instructions for authors in PLOS journals).

The results of the citation analysis performed in WoS, WoS CRS and GS are summarized in Table 13. Following indicators were used in WoS Core Collection: percentage of cited documents, total number of citations, citations per cited publication, maximum of citations, h- and m-indices, i10 (number of publications with at least 10 citations), and the number and percentage of Top 10 % and Top 1 % most cited publications calculated for each WoS category. The two last indicators related to the top most cited publications were not available in WoS CRS and GS, since these data sources do not provide reference citations values per subject category and year of publication. So these were substituted by different variations of the i-index (i10, i50, i100, and i100), according to the number of citations attracted in each source. Table 13 shows that the values of the main citation indicators might differ in absolute values in GS, WoS Core Collection and WoS Cited Reference Search. Nevertheless, they correspond very well overall and thus, they are all valid and applicable in bibliometric studies.

**Table 13:** Citation analysis in the three data sources for each candidate (Exercise 4)

Data source	Citation analysis	Mean	Median	Standard deviation	Confidence *T*
Web of Science Core Collection	Total documents	21.11	20.00	9.46	7.27
	Citable items (A/R/PP)	18.67	19	8.60	6.61
	# Cited documents	13	15	6.78	5.21
	% Cited	59.83 %	66.67 %	0.16	0.12
	Total citations	162.11	100	168.32	129.38
	Total citations A/R/PP	142.89	100	140.61	108.08
	% Self-citations	14.13 %	14.71 %	0.08	0.06
	Citations/cited pub	10.37	7.06	8.01	6.16
	Maximum citations	53	33	70.18	53.94
	h-index	5.44	6	2.50	1.92
	m-index	0.34	0.35	0.18	0.14
	i10	4.11	4	3.03	2.33
	# Top 10 %	2.56	1	2.83	2.18
	% Top 10%	8.62 %	7.14 %	0.09	0.07
	# Top 1 %	0.33	0	0.67	0.51
	% Top1%	1.18 %	0.00 %	0.02	0.02
Web of Science Cited Reference Search	Total documents	38.22	36.00	17.79	13.67
	# Cited documents	38.22	36.00	17.79	13.67
	# Cited not in the list	7.11	4.00	6.19	4.76
	Not assigned	0.56	0.00	0.96	0.73
	Total citations	240.44	152.00	221.23	170.06
	Citations/cited pub	5.59	4.22	3.60	2.76
	Maximum Citations	58.44	33.00	84.13	64.67
	h-index	6.67	7.00	2.79	2.14
	m-index	0.41	0.45	0.18	0.14
	i10 citations	5.56	6.00	4.00	3.08
	i50	0.78	0.00	1.47	1.13
Google Scholar	Total documents	58.78	55	27.23	20.93
	# Cited documents	38.33	37	21.01	16.15
	% Cited	62.29 %	65.45 %	0.12	0.09
	# Not in the list	11.89	9	9.63	7.40
	# Cited not in the list	5	3	4.81	3.70
	Not assigned	2	0	3.40	2.61
	Total citations	803.11	646	745.12	572.75
	Citations/cited pub	17.69	15.38	9.18	7.05
	Maximum Citations	192.67	95	306.24	235.39
	h-index	12.67	14	5.12	3.94
	m-index	0.75	0.79	0.30	0.23
	i10	15.89	19	9.89	7.60
	i50	3.11	3	2.18	1.68
	i100	1.00	0	1.70	1.31
	i1000	0.11	0	0.31	0.24

Last, but not least, Table 14 shows a detailed citation analysis (including the total and mean number of citations, and the number of citations per cited items) in Google Scholar for each candidate of Exercise 4 and for the three most cited publication types monographs, book chapters and journal articles.

**Table 14:** Detailed citation analysis GS for each candidate and the three most cited publication types (Exercise 4)

Statistical data	Monographs in GS								
	Items				Citations				
	# Total	# Not list	# Cited	% Cited	# Total	Mean^a^	# Max	# Self^b^	% Self^b^
Mean	1.89	0.00	1.78	95.83 %	39.67	19.90	26.89	8.00	22.92 %
Median	2	0	2	100.00 %	12	14.5	9	8	22.92 %
Stand. Deviation	0.99	n.a.	0.92	0.11	49.64	16.90	31.78	7.00	0.15
Confidence *T*	0.76	n.a.	0.70	0.09	38.16	14.13	24.42	5.38	0.11

Statistical data	Journal articles in GS								
	Items				Citations				
	# Total	# Not list	# Cited	% Cited	# Total	Mean^a^	# Max	# Self^b^	% Self^b^
Mean	22.56	1.44	18.33	79.34 %	509.11	25.07	183.11	20.50	18.86 %
Median	26	2	21	75.00 %	284	16	71	20.5	18.86 %
Stand. deviation	7.89	n.a.	7.26	0.11	526.30	24.84	309.37	13.50	0.13
Confidence *T*	6.06	n.a.	5.58	0.08	404.55	19.09	237.80	10.38	0.10

Statistical data	Book chapters in GS								
	Items				Citations				
	# Total	# Not list	# Cited	% Cited	# Total	Mean^a^	# Max	# Self^b^	% Self^b^
Mean	12.33	0.56	8.33	62.34 %	119.89	11.08	33.00	3.00	2.89 %
Median	5	0	4	68.18 %	61	9.2	27	3	2.89 %
Stand. deviation	11.12	1.07	8.76	0.23	175.68	7.67	35.03	3.00	0.03
Confidence *T*	8.54	0.82	6.73	0.17	135.04	5.89	26.92	2.31	0.02

^a^Cited mean = # citations/cited item

^b^Only calculated for two candidates

*n.a.* not applicable

Concerning the percentage of cited documents the results of the citation analysis are very similar to the ones reported for exercise 3. It is evident for this research field, that journal articles contribute most to the total impact assessed by the number of citations. The most collaborative candidates no. 2 and 8 also achieve the highest citation scores.

## Conclusions and discussion

This study illustrates the special situation of the Geography discipline with its foundations as a natural and a social science discipline as well as a discipline which covers and addresses cross-cutting topics. Since research initiatives and networks are commonly oriented towards external cooperation to other disciplines or to focused national or international networks, the respective publications are also positioned within these links. This observation is explored in the following three different subsets.

### Publication strategies

Differences between research fields related to natural and social sciences are clearly visible. While in natural science, publications in highly ranked international peer-reviewed journals is of highest importance, the publications within the social science domain refer often to reports, book-chapters and also monographs. However, higher productivity (number of publications per year) and citation counts are relativized when also considering the higher number of co-authors and percentage of self-citations.

The results hint at very heterogeneous and individual publication strategies considering the selection of adequate publication channels. This is even true within the same research fields. Rather heterogeneous and individual publication strategies regarding the number of co-authors and the percentage of single-authored publications have been revealed even within the same research field. Certainly authors with a higher number of co-authors are usually the most productive ones, which results in attracting the highest impact scores. Hence this study corroborates one more time the benefits of co-operation also in this discipline.

General publication strategies, especially those based on the impact factor, are still more evident in the fields related to the natural sciences but they are also getting increasingly important in fields related to the social sciences. This might also be attributed to the publication language. With social science research, it is much more common to publish in the native language German. The reasonable argument herein is, that the study objects are embedded in the cultural language environment, and consequently, the publication of the research findings have to be made available in particular to this targeted group or entity. In contrast, research publications in natural sciences focus increasingly on the international audience, and thus, the researchers are much more present at international conferences, contribute more commonly also in international networks. Consequently, this leads to a higher percentage of English publications in international highly ranked peer-reviewed journals. Nevertheless, it has been recognized by all researchers in the Geography discipline that research needs to be distributed through all channels in order to raise the awareness of the performed analysis and achieved results. This is also visible in many time-line investigations of bibliometric analysis.

### Publication channels and their impact

Journal Articles and Book Chapters are the most common publication channels within Geography. A research output analysis performed a posteriori in the Current Research Information System (CRIS) of the University of Vienna (http://bibliothek.univie.ac.at/ucris.html) for the Department of Geography and Regional Research (IfGR) during the years 2012, 2013 and 2014 can be summarized as follows: (a) almost one third of the publications are published as contributions in journals, another third as contributions in books and less than one tenth as monographs, (b) 60 % of the journal articles were indexed in peer review journals, (c) half of the journal articles were indexed in the Web of Science (Science Citation and Social Science Citation Indexes), and (d) 42.5 % of the items indexed in WoS were published in Q1-Journals according to their impact factor in the corresponding Journal Citations Reports Edition (Top 25 %). The results gained in this study are in very good agreement with this analysis performed in the CRIS of the University of Vienna. This suggests that the general observations discussed in this contribution can also be seen in the IfGR of the University of Vienna.

Monographs, Journal Articles (including Proceedings Papers) and Book Chapters are the most cited document types in the fields strongly related to the natural sciences as well as in the ones related to the social sciences, with a different distribution within these three types, however.

In social science Geography, other publication channels including also non-scientific or non-peer-reviewed sources, like Films, Policy Briefs, Newspapers and Special Issues are very usual, even if they do not attract a high number of citations. Working Papers are also a relevant document type and require special attention, since they can attract a considerable number of citations. All these publications are commonly written in German. Their frequency certainly depends on the addressed target group. Tracking these channels can be used in order to assess the societal impact of the research performance in these fields within the regional setting and how these studies are embedded in the surrounding, often local communities.

### Comparison between data sources

As the analysis has shown clearly, citation analyses for articles, monographs, book chapters and reports (working papers) should be performed separately and require the inclusion of complementary data sources. Otherwise relevant publications can easily be missed, resulting in wrong interpretations. The results show that no considerable differences are observed when using WoS or Scopus. The inclusion of the Cited Reference Search allows for a significant coverage and is more practicable in WoS than in Scopus.

The coverage, especially of books (almost 100 %), is much higher in GS and suggests the recommendation of this complementary data source, although it is still a “black box” from a data base perspective (no transparency, missing content information, etc.). In this study the accuracy of the citations in GS was very high (~95 %). Nevertheless further analyses are needed to reduce the noise of GS data in order to increase the significance of the results.

The values of the main citation indicators might differ in absolute values in GS, WoS, WoS Cited Reference Search and Scopus, but are comparable in terms of relative values. This fact suggest an “iceberg analogy”. The citation analysis in WoS and Scopus reveals only the ‘visible part’, which usually can be measured. However, the much bigger ‘invisible part’ remains inaccessible for these citation databases, but can be explored to some extent by Google Scholar. Still, only future methods, products and metrics will be able to surface the currently hidden parts of the iceberg.

## General conclusions

Peers have to be aware of blind spots in ‘citation analyses’ (e.g. ‘non cited’ document types and publications, manuscripts wrongly assigned to specific types) with potentially harmful consequences in evaluation exercises. This should, however, not result in the conclusion that such an analysis is not useful. In contrast, it should be stressed, that the bibliometric analysis is most important and useful, but still has to be handled with great care. As a consequence, it has to be stressed that citations can only be used as a proxy for impact (and not for the quality) of publications produced in the ‘publish or perish’ community (i.e. the scientists who are committed to publishing their results). One has to use these metrics with caution. High “numbers” cannot always be related to “high quality” science. For example, a really bad publication including possibly even wrong data might be cited very often as an example of a bad paper—but consequently receives high bibliometric values. Although it is an extreme example indeed, it demonstrates the necessity for cautiousness when dealing with—and interpreting—such bibliometric indices.

Last but not least, the scientific community does not only serve for academics. It comprises also teaching academics as well as representatives from government or industry, who rather use that cite scientific output directly. Furthermore, our society has become progressively informed (‘societal impact’). Unfortunately alternative metrics (like usage metrics and altmetrics) are still in their infancy (Kurtz and Bollen 2010; Priem et al. 2012; Gorraiz et al. 2014; Hammarfelt 2014) to measure the impact beyond citations and could not yet be applied to the described appointment procedures due to the current lack of available and reliable data. Nevertheless, the provided data are an important accompanying information source in any selection process.

## Footnotes

1 See http://www.excelfunctions.net/Excel-Confidence-T-Function.html.

## References

[CR1] Australian Research Council. (2012). *The excellence in research for Australia (ERA) initiative*. http://www.arc.gov.au/era/. Accessed January 5, 2015.

[CR2] Dikau, R., & Pohl, J. (2011). Hazards: Naturgefahren und Naturrisiken. In H. Gebhardt, R. Glaser, U. Radtke, P. Reuber (Eds.), *Geographie* (pp. 1115–1170). Verlag: Spektrum Akad.

[CR3] Gebhardt, H., Glaser, R., Radtke, U., & Reuber P. (Eds.) (2011a). Geographie (p. 1328). Verlag: Spektrum Akad. ISBN:978-3-8274-2816-5.

[CR4] Gebhardt, H., Glaser, R., Radtke, U., & Reuber, P. (2011b). Verschiedene Antworten auf die Frage nach der Geographie. In H. Gebhardt, R. Glaser, U. Radtke, & P. Reuber (Eds.), *Geographie* (pp. 49–70). Verlag: Spektrum Akad.

[CR5] Glaser, R., & Gebhardt, H. (2011). Globaler Umweltwandel – Globaliserung – globale Ressourcenknappheit. In H. Gebhardt, R. Glaser, U. Radtke & P. Reuber (Eds.), *Geographie* (pp. 1171–1304). Verlag: Spektrum Akad.

[CR6] Gorraiz, J., & Gumpenberger, C. (2015). Exploration of the bibliometric coordinates for the field of geography. In A. A. Salah, Y. Tonta, A. A. Akdag Salah, C. Sugimoto, & U. Al (Eds.), *Proceedings of ISSI 2015 Istanbul: 15th International Society of Scientometrics and Informetrics Conference, Istanbul, Turkey*, 29 June to 3 July, 2015, Bogaziçi University Printhouse, pp. 139–149.

[CR7] Gorraiz J, Gumpenberger C (2015). A bibliometric model for the enhancement of professorial appointments. Scientometrics.

[CR8] Gorraiz J, Gumpenberger C, Schlögl C (2014). Usage versus citation behaviours in four subject areas. Scientometrics.

[CR9] Gorraiz J, Purnell PJ, Glänzel W (2013). Opportunities for and limitations of the book citation index. Journal of the American Society for Information Science and Technology.

[CR10] Hammarfelt B (2014). Using altmetrics for assessing research impact in the humanities. Scientometrics.

[CR11] Harzing, A. W. (2007). *Publish or perish*. http://www.harzing.com/pop.htm.

[CR12] Jacso P (2005). Google scholar: The pros and the cons. Online Information Review.

[CR13] Kousha K, Thelwall M (2007). Google Scholar citations and Google Web/URL citations: A multi-discipline exploratory analysis. Journal of the American Society for Information Science and Technology.

[CR14] Kousha K, Thelwall M (2009). Google book search: Citation analysis for social science and the humanities. Journal of the American Society for Information Science and Technology.

[CR15] Kurtz MJ, Bollen J (2010). Usage bibliometrics. Annual review of information science and technology.

[CR16] Labbé C, Labbé D (2013). Duplicate and fake publications in the scientific literature: How many SCIgen papers in computer science?. Scientometrics.

[CR17] López-Cózar ED, Robinson-García N, Torres-Salinas D (2014). The Google Scholar experiment: How to index false papers and manipulate bibliometric indicators. Journal of the Association for Information Science and Technology.

[CR18] Meho LI, Yang K (2007). Impact of data sources on citation counts and rankings of LIS faculty: Web of science versus scopus and google scholar. Journal of the American Society for Information Science and Technology.

[CR19] Moksony F, Hegedűs R, Császár M (2014). Rankings, research styles, and publication cultures: a study of American sociology departments. Scientometrics.

[CR20] Nederhof AJ (2006). Bibliometric monitoring of research performance in the social sciences and the humanities: A review. Scientometrics.

[CR21] Ossenblok TLB, Engels TCE, Sivertsen G (2012). The representation of the social sciences and humanities in the Web of Science—A comparison of publication patterns and incentive structures in Flanders and Norway (2005–9). Research Evaluation.

[CR22] Priem, J., Piwowar, H. A., & Hemminger, B. M. (2012). *Altmetrics in the wild: Using social media to explore scholarly impact*. arXiv preprint:1203.4745. http://arxiv.org/abs/1203.4745. Accessed Jan 5, 2015.

[CR23] van Leeuwen, T. N. (2013). Bibliometric research evaluations, Web of Science and the Social Sciences and Humanities: A problematic relationship? *Bibliometrie*-*Praxis and Forschung, 2*. http://www.bibliometrie-pf.de/article/viewFile/173/218. Accessed Jan 5, 2015.

